# Alginate-like polymers from full-scale aerobic granular sludge: content, recovery, characterization, and application for cadmium adsorption

**DOI:** 10.1038/s41598-022-26743-5

**Published:** 2022-12-23

**Authors:** Agnieszka Cydzik-Kwiatkowska, Mariusz Z. Gusiatin, Magdalena Zielińska, Irena Wojnowska-Baryła, Dorota Kulikowska, Katarzyna Bernat

**Affiliations:** Department of Environmental Biotechnology, Faculty of Geoengineering, University of Warmia and Mazury in Olsztyn, Słoneczna St. 45G, 10-709 Olsztyn, Poland

**Keywords:** Environmental sciences, Environmental chemistry, Environmental impact

## Abstract

Aerobic granular sludge (AGS) is a proven resource for the recovery of biopolymers like alginate-like polymers (ALP). This is the first report on the dynamics of ALP produced by AGS (ALP-AGS) in a full-scale wastewater treatment plant (WWTP), optimization of ALP recovery from AGS, and adsorption of cadmium (Cd^2+^) by ALP. Recovery of ALP was highest when using 120 mL of 0.2 M Na_2_CO_3_ at 70 °C for 45 min. Seasonal (1.5 years, over 3100 cycles) and intra-cycle changes in ALP-AGS in the WWTP were monitored. The ALP content in AGS increased in the transition period between winter and spring, reaching over 150 mg/g MLSS. In the batch reactor cycle, the ALP-AGS level peaked 2 h after the start of aeration (mean peak level: 120 mg/g MLSS), then decreased about two-fold by the end of the cycle. The ALP-AGS had a small surface area and a lamellar structure with crystalline outgrowths. The optimal conditions of Cd^2+^ adsorption with ALP were a dosage of 7.9 g d.m./L, a pH of 4–8, and an equilibrium time of 60 min. Carboxyl and hydroxyl groups were the key functional groups involved in Cd^2+^ adsorption. According to the Sips model, the maximum Cd^2+^ adsorption capacity of ALP-AGS was 29.5 mg/g d.m., which is similar to that of commercial alginate. AGS is a richer source of ALP than activated sludge, which ensures the cost-effectiveness of ALP recovery and increases the sustainability of wastewater treatment. Information on the chemical properties and yields of ALP from full-scale WWTPs is important for downstream applications with the recovered ALP.

## Introduction

Aerobic granular sludge (AGS) technology has been widely applied in full-scale wastewater treatment plants (WWTPs). Field reports indicate that AGS enables more stable and efficient wastewater treatment than conventional activated sludge due to higher biomass concentrations in the reactors, better resistance to unfavorable environmental conditions, and more effective sludge separation from the treated wastewater^1^. During granulation, operational parameters are selected to favor the production of extracellular polymeric substances (EPS), which support bacterial aggregation and form a matrix that encapsulates and protects the bacteria^2^. EPS have a critical functional role in the formation and stabilization of sludge granules, influencing the granules’ structure, settling performance, surface charge, flocculation, and dewatering properties^3^. EPS consist of a very complex mixture of biomolecules (proteins, humic-like substances, polysaccharides, uronic acid, nucleic acid, lipids, glycoproteins), which can be excreted by microorganisms, produced from cell lysis and hydrolysis, and adsorbed from wastewater^4–6^. Extracting EPS from excess AGS reduces the amount of sludge discarded by 20–35%^7^ and thus significantly decreases the amount of sludge that requires management. After EPS extraction, waste sludge has better biodegradability, making anaerobic digestion of the sludge more efficient^8^.

The main fraction of EPS is alginate-like exopolymers (ALE), which are formed not only by polysaccharides, but also by other polymers such as proteins, humic substances, and lipids^9^. During ALE extraction, some intracellular polysaccharides and proteins can be released by cell lysis^10^; this combination of ALE and intracellular polymers is referred to as alginate-like polymers (ALP)^9^.

Although there are reports on the effect of environmental conditions on ALP production in pure bacterial cultures, studies of ALP production in wastewater treatment systems are scarce. The extraction and recovery of ALP from AGS have attracted great interest. Currently, most extractions and purifications of ALP are performed with AGS, while fewer studies have investigated the recovery of ALP from activated sludge^11^. This is because the content of ALP is higher in AGS than in activated sludge^12^. Extractable ALP can account for up to 25% of the mass of AGS^13^. The production of ALP in an AGS system depends on many factors; thus, to ensure highly effective ALE recovery, the operating parameters must be optimized. In lab-scale reactors fed with wastewater containing propionate and acetate, the biomass contained significantly more ALP (up to 261 ± 33 mg volatile solids (VS)/g VS sludge) than the biomass in a reactor fed with volatile-fatty-acid–free wastewater. dos Santos et al., reported that the highest content of biopolymers (418.7 mg ALP/g VSS) was found in AGS in acetate-fed reactors. The production of ALP was also significantly higher when propionate was used as the substrate than when glycerol, glucose, or sucrose were used^14^. The composition of ALP was similar in activated sludge flocs and aerobic granules^9^. ALP production was highest when a lab-scale AGS reactor was operated with a short anoxic phase in the anaerobic/oxic/anoxic cycle and a short solid retention time of about 10 days^15^. Increasing the COD:N ratio increased the amount of ALP in the biomass, but when the ratio reached 30:1, ALP production decreased^11^. de Carvalho et al.^16^ indicate that the conditions for ALP production are most favorable in reactors with mature AGS that are operated with a variable organic load, a long sludge retention time (up to 20 days), a COD/N ratio of 20, and moderate salinity of the influent wastewater. Many studies on the production and utilization of ALP still focus on the use of synthetic influents, which do not truly represent the applicability of the system on a real scale^12^. In a study conducted in a pilot-scale WWTP with AGS treating municipal sewage containing approximately 25% slaughterhouse wastewater, the yield of extractable ALE reached 160 ± 4 mg/g VSS. The ALE were characterized by a high percentage of poly-guluronic acid blocks and formed rigid, non-deformable gels in CaCl_2_^17^.

Due to the complex structure of ALP, it is practically impossible to extract all of their components with a single method. Various physical methods (e.g., centrifugation, sonication, heating) and chemical methods (extraction with formamide, NaOH, EDTA, Na_2_CO_3_) have been used to extract and recover polymers from AGS^13,18^. The choice of extraction method affects not only the total amount of polymers obtained but also their composition. Currently, the most suitable technique for extracting ALP is alkaline extraction using sodium carbonate (Na_2_CO_3_) at high temperatures^16^, which is similar to the extraction of alginate from brown algae. When dealing with tightly bound extracellular polymers in AGS, ALP extraction and recovery can be improved by acidic, microwave, or ultrasonic pretreatment^18^.

Polymers recovered from waste AGS can be used to produce flame retardants, absorbing gels, ink thickeners, gluing agents for fertilizer pellets, corrosion inhibitors, coatings for improving the water resistance of paper, or fire-resistant boards^19–21^. The structural fraction of EPS (sEPS) can be extracted and recovered from AGS and used to form hydrogels via a cross-linking process employing Ca^2+^ solutions. These hydrogels have a water-holding capacity of up to 99 gH_2_O/g sEPS and can be used in the chemical, paper, textile, or agronomic industrial sectors^22^. ALP recovered from AGS provide sorption sites for various compounds^23^ like methylene blue^24^ and for phosphorus^25^ and heavy metals (Pb^2+^, Cu^2+^, Ni^2+^, and Zn^2+^)^26,27^. Cd^2+^ is a priority pollutant due to its extreme toxicity and adverse effects on human health, and it should be eliminated from the environment. A previous study confirmed that Cd^2+^ was very effectively removed from water by EPS extracted from excess activated sludge from a municipal WWTP after short-duration aerobic digestion^28^.

ALP are regarded as one of the most promising bioproducts that can be recovered from WWTPs^29^. So far, no investigations have been done regarding the effect of seasonal and intra-cycle changes on ALP content in AGS from full-scale WWTPs. Defining the best periods for recovery of biopolymers from AGS may ensure high polymer yields and favor the economy of the process in full-scale applications. Moreover, the sorption potential of ALP recovered from AGS from a full-scale system has also not been tested in the context of heavy metal removal. Therefore, the objectives of this study were to optimize the extraction of ALP from AGS, to monitor the content of ALP in AGS during granular batch reactor cycles and seasonal temperature variations in a large-scale wastewater treatment plant, and to characterize the ALP and their ability to adsorb Cd^2+^ from aqueous solutions. The content of ALP in AGS was monitored for 1.5 years in a full-scale WWTP treating municipal wastewater. This is the first time that a potential valorization strategy for waste AGS has been studied in a full-scale technological system. Knowledge about the yield and chemical characteristics of the obtained ALP is important because these features are essential for downstream applications.

## Materials and methods

**Figure 1:**
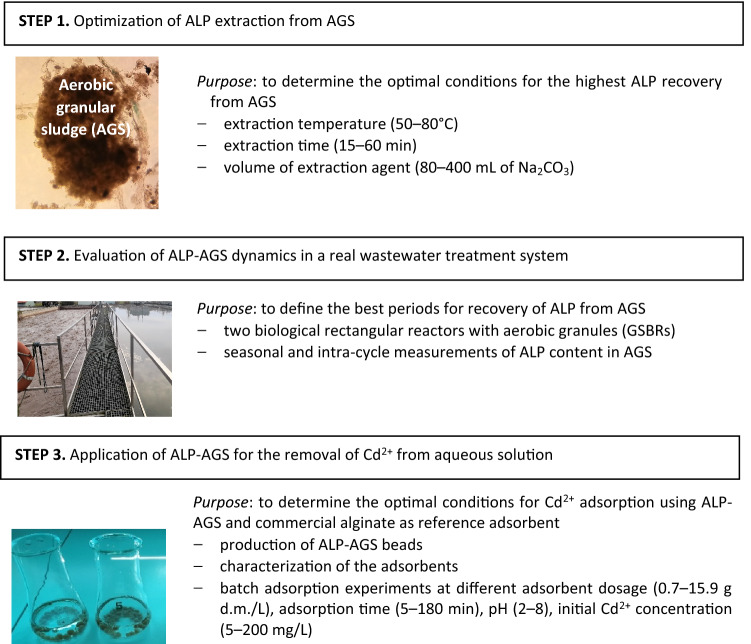
Flowchart of the experimental setup.

The experiments were performed in 3 steps: (1) optimization of ALP extraction from AGS, (2) evaluation of ALP-AGS dynamics in a real wastewater treatment system and (3) application of ALP-AGS for the removal of Cd^2+^ from aqueous solution. The flowchart of the experimental setup is shown in Fig. 1.

### AGS sampling

Granular sludge was collected from WWTP in Lubawa (Poland). This plant operates at a low organic load and uses AGS technology to treat wastewater in amounts corresponding to 15,000 PE (population equivalent). The average wastewater flow is about 3200 m^3^/day; about 30–40% of the influent is wastewater from the dairy industry. The volumetric loading rate of the WWTP is 0.77 m^3^/(m^3^ day). The wastewater contained about 1395 ± 65 mg COD/L, 18.1 ± 1.3 mg TP/L, 90.7 ± 15.2 mg TN/L, and 55.8 ± 20.0 mg N_org_/L. The technological line of the WWTP consisted of a stepped grid, grit chamber, flow equalization tank, two identically operated biological rectangular reactors with aerobic granules (i.e. GSBRs), a tank equipped with a stirrer, where phosphorus precipitation can be performed if necessary, and four secondary clarifiers, from which the effluent was discharged into a river. Waste (excess) sludge from GSBR was aerobically stabilized. The stabilized sludge and the waste sludge from the secondary clarifiers were collected in a sludge thickener and then dewatered using a mechanical belt thickener.

The cycle of a biological reactor begins with simultaneous, piston feeding of raw wastewater and withdrawal of effluent. The whole reactor cycle lasted for 4.8 h and consisted of 0.8 h of anoxic filling with simultaneous wastewater withdrawal, an aeration period of 3.4 h, and 0.6 h of settling and biomass discharge. The volumetric exchange rate was about 25%. The AGS was sampled from February 2018 to August 2019 from both GSBRs at the end of the aeration phase in the cycle (every 25 days on average). Intra-cycle measurements of ALP content in AGS were performed in two independently operated full-scale GSBRs during the aeration phase of cycle 916th. Before analysis, the sludge was stored at 4 °C for no longer than 24 h. In the investigated period, the average biomass concentrations were 6.8 g MLSS/L (GSBR#1) and 7.0 g MLSS/L (GSBR#2). The sludge volumetric index in both GSBRs was at a level of 50 mL/g MLSS.

### ALP extraction from AGS

The content ALP in AGS was measured according to Lin et al.^17^, but the procedure was optimized to ensure the highest yield of ALP from AGS. Therefore, 2.5 g of MLSS was homogenized using Ultra Turrax T25 basic (IKA-WERKE) for 5 min at 9.500 1/min, and then incubated from 15 to 60 min at temperatures varying from 50 to 80 ºC in 0.2 M Na_2_CO_3_—a volume of solution from 80 to 400 mL. Biomass was then centrifuged at 12,000 rpm for 20 min. The reaction of the supernatant was adjusted to pH 2 using 0.1 M HCl. The supernatant was centrifuged at 12,000 rpm for 20 min and the ALP was dissolved in 0.1 M NaOH. ALP was precipitated using cold EtOH adjusting the solution to 80% (v/v), lyophilized, and weighed. The extracted ALP was used in further experiments.

### Adsorbent preparation

Cd^2+^ adsorption experiments were performed with ALP-AGS which was in the form of beads. Sodium alginate (ALG) purchased from Sigma-Aldrich was used as a reference adsorbent. The beads were prepared by dropwise addition of a viscous 2.5% (w/w) ALG or ALP-AGS aqueous solution to 0.03 M CaCl_2_ solution with the help of an injection syringe. The gelation process was continued for 30 min. During the gelation process, the alginate reacts with the Ca^2+^ ions from the CaCl_2_ solution to form a cross-linked Ca-alginate. The obtained beads were left in the solution for 24 h, and then kept in distilled water at 4 °C in the refrigerator. The experiments on Cd^2+^ adsorption were performed with fresh beads.

### Batch adsorption experiments

Commercial stock standard Cd^2+^ solution at a concentration of 1000 mg Cd^2+^/L (Sigma-Aldrich) was used. Working standards of required concentration were prepared by diluting the stock solution in distilled water.

Cd^2+^ removal from aqueous solution was conducted at room temperature, in a function of adsorbent dosage (0.7, 2.6, 5.3, 7.9, 10.6, 13.2, and 15.9 g d.m./L), contact time (5, 15, 30, 60, 90, 120, 150, 180 min.), pH (2, 3, 4, 5, 6, 8) and initial Cd^2+^ concentration (5, 10, 15, 30, 50, 75, 100, 150, 200 mg/L). The experiments were conducted in 125-mL Erlenmeyer flasks. The adsorbent dosage for pH and contact time experiments was set to 2.6 g d.m./L, while for the effect of initial Cd^2+^ concentration it was fixed to 7.9 g d.m./L. The pH of the Cd^2+^ working solution in the experiments, except for the effect of pH, was adjusted to 5.0 ± 0.2 using 0.1 M HNO_3_ or 0.1 M NaOH. The samples were agitated on a Gerhard shaker at room temperature water bath at 120 rpm for 2 h. The solutions thus obtained were filtered through a 0.45 µm filter and analyzed for Cd^2+^ concentration. Before metal analysis, the supernatants were acidified with HNO_3_. The residual adsorbent from the sorption experiment at selected conditions was dried to constant mass at 70 °C and characterized with advanced imaging and spectroscopic techniques.

### Analytical methods

Total Cd^2+^ concentration was measured with a flame atomic absorption spectrometer (FAAS) (Varian, AA28OFS) at 228.8 nm. The accuracy of the Cd^2+^ analysis was validated with the reference material (CRM 142 R). The limit of Cd^2+^ detection (LOD) was 0.07 mg/L, while the limit of Cd^2+^ quantification (LOQ) was 0.21 mg/L. The functional groups on the surface of ALP-AGS and ALG, before and after Cd^2+^ sorption, were analyzed in the range of 3800–400/cm using an FTIR spectroscope (Nicolet 6700, Thermo Scientific) equipped with a Smart Multi-Bounce HATR™. The surface morphology of adsorbents was examined with an LEO 1430VP scanning electron microscope (SEM) (Carl Zeiss). Qualitative and quantitative analysis of elemental surface composition (SEM–EDX) was performed with an energy-dispersive X-ray spectrometer (EDX, Quantax 200; detector: XFlash 4010, Bruker AXS, Berlin, Germany). Elemental mapping was performed on different micro-areas on the surface of ALP-AGS and ALG and the content of each element (in mass %) was averaged.

The Brunauer-Emmet-Teller (BET) specific surface area of adsorbents was determined by fitting the BET equation to the linear portion of the BET plot; the pore size distribution was calculated on the basis of the desorption plot of the N_2_ adsorption–desorption isotherm using the Barret-Joyner-Halenda method (Micrometrics ASAP 2000, USA).

### Calculations

The amount of Cd^2+^ adsorbed onto the adsorbent in the equilibrium (*q*_*e*_, mg/g d.m.) was calculated according to the following formula:

1 $$ q_{e} = \, ((C_{0} {-}C_{e} )/m) \cdot V $$

The efficiency (%) of Cd^2+^ adsorption was calculated according to the following formula:

2 $$ efficiency = \, ((C_{0} {-}C_{e} )/C_{0} ) \cdot {1}00\% $$

where *C*_*0*_ is the initial Cd^2+^ concentration in the solution (mg/L), *C*_*e*_ is the equilibrium Cd^2+^ concentration (mg/L), *m* is the mass of adsorbent (g d.m.), and *V* is the volume of metal solution (L).

### Equilibrium isotherm models

Three models were employed to interpret the adsorption isotherm data onto tested adsorbents:

3 $$ {\text{Freundlich}}\;{\text{model}}:\;q_{e} = K_{F} \cdot C_{e}^{1/n} $$

4 $$ {\text{Langmuir}}\;{\text{model}}: \;q_{e} = \, \left( {q_{max} \cdot b \cdot C_{e} } \right)/\left( {{1} + b \cdot C_{e} } \right) $$

5 $$ {\text{Sips}}\;{\text{model}}:\; q_{e} = \, \left( {q_{max} \cdot b \cdot C_{e}^{n} } \right)/\left( {{1} + b \cdot C_{e}^{n} } \right) $$

where: Freundlich model: *K*_*F*_ is the constant in the Freundlich equation (L/mg), 1/*n* is a measure of adsorption intensity (–); Langmuir model: *q*_*max*_ is the maximum monolayer adsorption capacity (mg/g d.m.), *b* is adsorption equilibrium constant (L/mg); Sips model: *b* is Sips isotherm constant related to the energy of adsorption (L/mg), *n* is a constant related to the grade of surface heterogeneity (–).

### Kinetics model

The pseudo-second-order kinetic model was used to describe the kinetics of Cd^2+^ adsorption onto adsorbents:

6 $$ q_{t} = q_{e} {-} \, \left( {q_{e} /{1} + q_{e} \cdot k \cdot t} \right) $$

where: *q*_*t*_ is the amount of Cd^2+^ adsorbed at a specific adsorption time (mg/g d.m.); *k* is a rate constant (g d.m./(mg min)), and *t* is the adsorption time (min). The initial adsorption rate (*r*) was calculated as *k*‧*q*_*e*_^*2*^.

The fitting of the adsorption and kinetics isotherms to experimental data and the adsorption (*K*_F_, *q*_max_, *b*, *n*) and kinetics parameters (*q*_e_, *k*) were calculated by the Levenberg–Marquardt optimization method included in the STATISTICA® software (version 13.3, TIBCO Software Inc.). The appropriateness of the adsorption and kinetic isotherm models was determined based on the sum of squared errors (SSE) and coefficient of determination (R^2^).

## Results and discussion

### Optimization of ALP recovery from AGS

**Figure 2:**
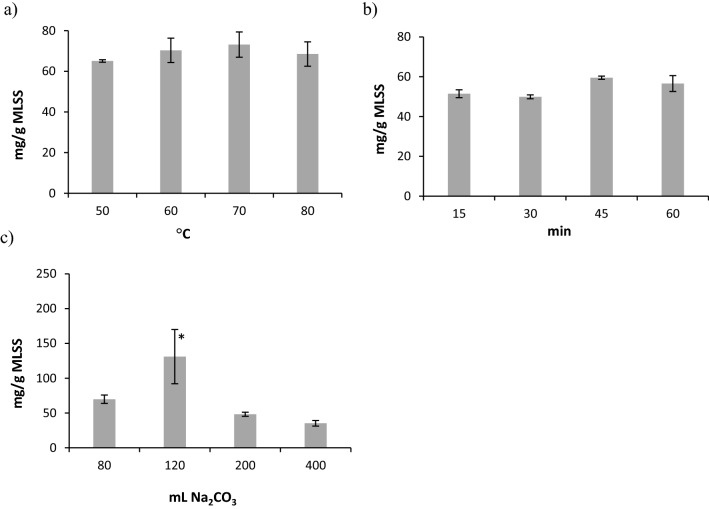
Yields of ALP-AGS (expressed as mg TS ALP/g MLSS) at different extraction conditions: (**a**) temperature, (**b**) time, (**c**) volume of Na_2_CO_3_; n = 2; *—significant difference compared to other volumes of Na_2_CO_3_ (ANOVA, Tukey’s HSD test, p = 0.05).

The yield of ALP-AGS was highest with an extraction temperature of 70 °C (Fig. 2a), an extraction time of 45 min (Fig. 2b), and 120 mL of 0.2 M Na_2_CO_3_/2.5 g MLSS (131 mg/g MLSS; significant differences, ANOVA, Tukey’s HSD test, p = 0.05) (Fig. 2c). This time was shorter and the temperature of extraction was lower than in the original methodology presented by Lin et al.^17^, which creates some opportunities for decreasing the costs of ALP recovery from AGS in full-scale installations.

### Seasonal and intra-cycle changes in ALP content in AGS

**Figure 3:**
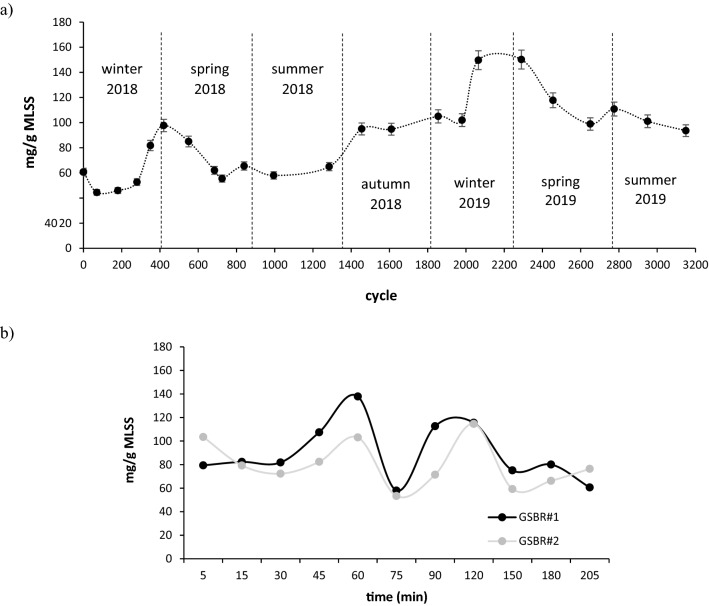
Variations in ALP content in AGS; (**a**) seasonal (n = 2, two independently operated full-scale GSBRs), (**b**) intra-cycle (measurement performed in two independently operated full-scale GSBRs during the aeration phase of the cycle 916).

The concentration of ALP-AGS was investigated for over 3100 cycles of stable performance in two independently operated full-scale GSBRs at a municipal WWTP. In this period, no serious operational problems were reported, and wastewater composition did not vary significantly. Both GSBRs were operated at similar biomass concentrations, which caused the main operational parameters of the GSBRs, such as the organic loading rate, to be nearly identical. At the respective beginnings of spring 2018 and spring 2019, the ALP-AGS content reached nearly 100 mg/g MLSS and over 150 mg/g MLSS (Fig. 3a). These values were slightly higher than the ALP-AGS content reported in another study conducted in a full-scale WWTP^30^ and similar to values observed in lab-scale reactors, which are usually higher than in full-scale systems^23^. For example, in lab-scale reactors fed with primary effluent from municipal wastewater, AGS contained 184 ± 18 mg VS ALP/g VSS^9^.

In this study, the ALP-AGS concentration in sludge increased significantly in the transition periods between winter and spring. During these periods, the temperature dropped to 9 °C and was the lowest of the entire experimental period (Fig. S1, Supplementary Materials). Bacteria may produce EPS as a strategy for survival in cold environments, as the presence of EPS significantly reduces cell lysis^31^. In pure strains of *Lactobacillus paracasei*, low temperatures increased the content of the high molecular weight fraction of EPS and the total amount of EPS produced^32^. Our results indicate that low temperatures also affect the ALP content in AGS—the highest yields of this biopolymer from waste sludge at the WWTP can be recovered at the end of winter. The average concentrations of ALP in biomass were higher in 2019 than in 2018; this may be explained by gradual granule maturation, which favors biopolymer production^33,34^. In a study by Huang et al.^35^, ALP were only found in mature granules.

Variations in the amounts and characteristics of ALP (mostly the molecular weight, MW) during the operational cycle of industrial-scale batch reactors have been reported^36^. In the present study, the amount of ALP varied considerably during the cycle, from about 50 to over 120 mg ALP/g MLSS, and two peaks of ALP content in biomass were observed, 1 and 2 h from the beginning of aeration (Fig. 3b).

ALP production has been reported to follow almost the same trend as bacterial growth^37^. Similarly, in the present study, the peak activity of bacteria, as indicated by the 16S rRNA levels in cells (results not shown), overlapped with the peaks of ALP production. During the introduction of wastewater to the reactors, the dissolved oxygen concentrations in the bulk liquid dropped significantly to below 0.5 mg/L in the GSBRs. Such low oxygen concentrations in the environment stimulate anoxic and anaerobic metabolism in the middle and core granule layers^38^. Therefore, the first peak in ALP formation may have resulted from anaerobic bacteria, such as phosphate-accumulating and glycogen–accumulating microorganisms, intensively producing ALP after the start of aeration^15^. As alginate contains glucuronic acid, it can act as a barrier to oxygen diffusion, limiting its transfer to the enzyme complexes in bacterial cells and protecting the metabolic activity of strict anaerobes^39^.

In the present study, ALP production started to increase in the 75th minute of the GSBR cycle and peaked at 2 h, which can be explained by an increase in ALP biosynthesis after the depletion of easily biodegradable acetate present in the wastewater introduced to the GSBR (data not shown). This explanation is consistent with a report of reduced ALP production in bacterial cells after the addition of sodium acetate to pure cultures of *Azotobacter vinelandi* cultivated on glucose-based media^40^. A similar pattern of ALP production has been reported in lab-scale reactors operated with an anaerobic/oxic/anoxic cycle and fed with wastewater containing sodium acetate^15^. In that study, ALP content was highest after 90 min of aeration. In the present study, the reduction in ALP levels in AGS at the end of the GSBR cycle can be explained by ALP lyase activity, which degrades ALP in the post-polymerization step^41^. This reduction in ALP levels is consistent with reports of batch tests, in which ALP production and its molecular weight dropped over time^42,43^. Practically speaking, our results indicate that, in a full-scale operation aimed at ALP recovery, the sludge must be discharged about 2 h after the start of aeration.

### Adsorbent characteristics

ALP-AGS and ALG had a low surface area (Table 1). The pore volume of ALP-AGS was about 24 times larger than that of ALG, but both adsorbents had similar pore diameters, the size of which indicated that they had microporous structures^44^. The surface area of ALG can vary considerably, depending on ALG conditions (e.g., the sodium alginate concentration, the type and concentration of gelation agent, etc.) or ALG modification. ALP-AGS that was produced with much more concentrated CaCl_2_ (12.5%) than that in the present study had a much larger surface area (76.2 m^2^/g) and a slightly larger pore volume (0.0623 cm^3^/g), but the pores had a smaller diameter (0.0177 nm)^25^. Aziz et al.^45^ have shown that the surface area of pristine ALG was low (4.1 m^2^/g), but that it increased 76 times (on average) after ALG modification with natural clay, natural phosphate, or activated charcoal. Because the aim of our preliminary study was to compare the usability of waste ALP with commercial ALG for metal removal, both adsorbents were used without any modification.

**Table 1:** Basic characteristics of the adsorbents (mean ± SD, n = 3).

Characteristics	Unit	ALG	ALP-AGS
Diameter of beads^a^	mm	2.7 ± 0.2	2.9 ± 0.3
Dry matter	%	5.3 ± 0.1	2.7 ± 0.3
Organic matter	% d.m.	74.4 ± 0.4	87.0 ± 1.6
BET surface area	m^2^/g	1.39 ± 0.06	2.08 ± 0.04
Pore volume	cm^3^/g	0.0013	0.031
Average pore diameter	nm	2.70	2.72

^a^Diameter of adsorbent beads after gelation.

### Structural morphology

**Figure 4:**
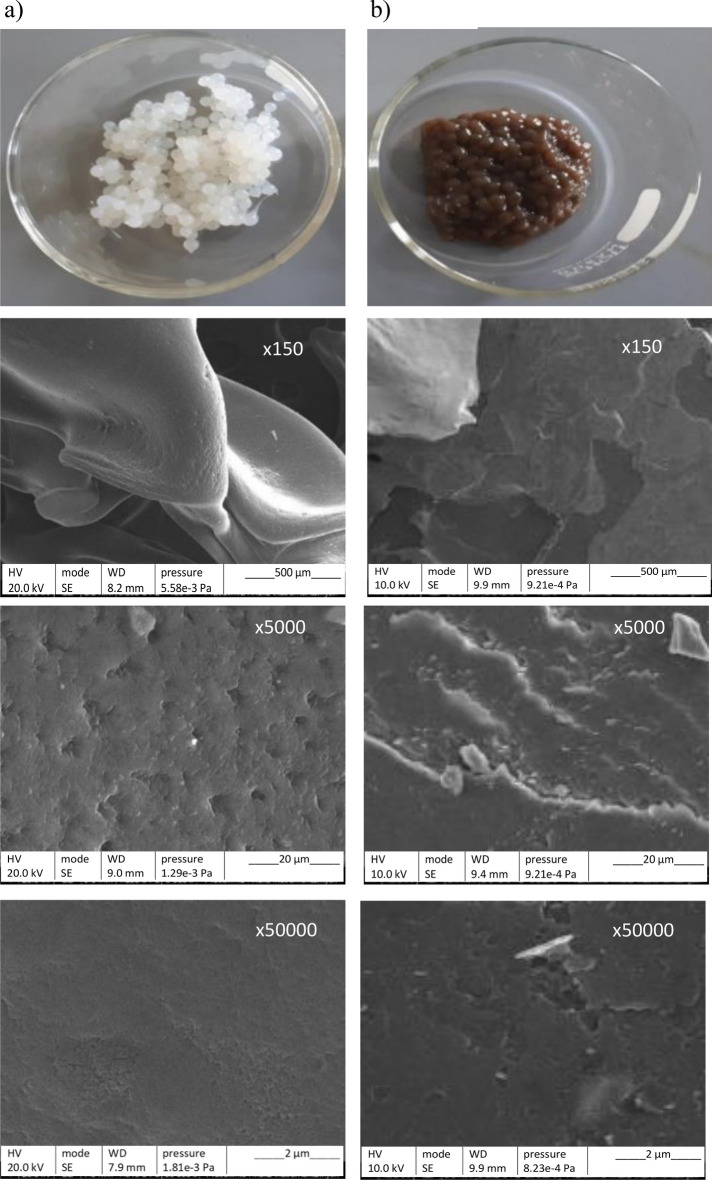
Adsorbent beads and their respective SEM micrographs at different magnifications (× 150, × 5000, × 50,000): (**a**) ALG, (**b**) ALP-AGS.

SEM images of the surface of ALG and ALP-AGS (Fig. 4) showed that both adsorbents had a very dense appearance without much porosity, which can indicate strong cross-linking caused by Ca^2+^ ions during the reaction with CaCl_2_^46^. Similarly, Torres-Caban et al.^47^ used SEM analysis to find that calcium alginate beads had a smooth surface without much porosity. At higher magnifications, the surface of the ALP-AGS was less regular and contained structures resembling crystalline outgrowths. An irregular and rough surface might indicate strong cross-links with Ca^2+^^34^ and can be also attributed to water evaporation and surface shrinking^48^.

### Elemental composition

SEM–EDX analysis revealed 9 elements on the ALG surface, and the major constituents (expressed in mass %) were C (11.03 ± 0.08), O (67.76 ± 4.20), Ca (12.53 ± 0.04), Na (3.95 ± 0.11), and Cl (2.97 ± 0.15) (Fig. S2). On the ALP-AGS surface, 12 elements were identified, with lower contents of C (5.48 ± 2.28), O (58.74 ± 1.70), and Na (0.96 ± 0.21) than ALG, and higher contents of Ca (13.04 ± 2.88), Al (1.87 ± 0.19), S (1.29 ± 0.25), P (4.04 ± 0.30), and Cl (6.31 ± 0.24). In contrast to ALG, ALP-AGS also contained N (7.47 ± 0.23) and trace amounts of Fe (0.16%) and Cu (0.30%). For comparison, Isik et al.^49^ showed that calcium ALG beads were composed of C (9 mass %), O (19 mass %), Na (6 mass %), Cl (34 mass %), and Ca (32 mass %). The presence of C and O may correspond to functional groups distributed on the surface of the adsorbents^50^. The presence of Cl in alginate sorbents is often due to incomplete washing of the adsorbent with water at the end of gelation with CaCl_2_. The presence of S and Al in ALG beads can be due to algae components or impurities associated with alginate extraction^47^. Finally, the nitrogen and phosphorous in the ALP-AGS indicate that these nutrients were transferred from AGS during ALP isolation.

### Adsorption of Cd^2+^ onto ALG and ALP-AGS

Cd^2+^ adsorption onto the tested adsorbents was optimized in terms of adsorbent dosage, pH, initial Cd^2+^ concentration, and sorption time.

### Adsorbent dosage

**Figure 5:**
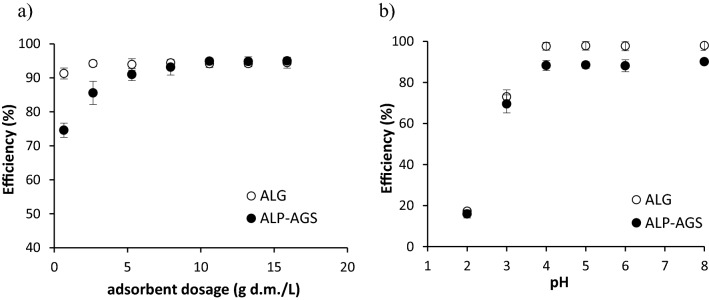
The efficiency of Cd^2+^ adsorption onto ALG and ALP-AGS depending on: (**a**) adsorbent dosage (10 mg Cd^2+^/L; pH 5.0; 2 h); and solution pH (10 mg Cd^2+^/L; 2 h; adsorbent dosage 2.6 g d.m./L); n = 2.

At the lowest adsorbent dosage (0.7 g d.m./L), the process efficiency was higher for ALG (91%) than for ALP-AGS (75%) (Fig. 5a). This may be connected with the lower number of active sites on ALP-AGS. As the dosage of ALP-AGS was increased from 0.7 to 7.9 g d.m./L, the efficiency increased from 75 to 93%, which can be attributed to the presence of a greater number of adsorbent sites at a higher adsorbent dosage^50^. With adsorbent dosages in the range of 7.9–15.9 g d.m./L, the average Cd^2+^ adsorption efficiency, at an initial metal concentration of 10 mg/L, was 94.3 ± 0.2% for ALG and 94.4 ± 0.9% for ALP-AGS. Based on the residual concentration of Cd^2+^ in the solution after adsorption and the Cd^2+^ removal efficiency, an ALP-AGS dosage of 7.9 g d.m./L was selected as optimum.

### pH

The efficiency of Cd^2+^ removal by ALG and ALP-AGS depended on the solution pH (Fig. 5b). The pH is known to be important for controlling metal adsorption, as it affects both the chemical properties of surface functional groups and the speciation of metal ions^51^. The best sorption effects with both sorbents were obtained with pH values in the range of 4–8; at these values, the average adsorption efficiency was 88.7 ± 0.9% for ALP-AGS and 97.0 ± 0.2% for ALG. Adsorption efficiency and adsorption capacity were lowest at pH 2–3. Kuczajowska-Zadrożna et al.^52^ found that Cd^2+^ adsorption onto ALG beads reached 91% at pH values ranging from 5.0 to 9.0, and that reducing the pH to 2.0 significantly decreased the adsorption efficiency to 23%. Mahmood et al.^53^ reported that pH 6.0 was optimum for Cd^2+^ adsorption onto ALG.

Changes in the pH can affect the charge of functional groups on the surface of the adsorbent, which, in turn, affects its capacity to adsorb metals. ALG sorbents consist mainly of guluronic and mannuronic acid units containing carboxyl and hydroxyl groups^54,55^. The pKa values of the carboxyl groups of the mannuronic and guluronic acid units in ALG are 3.38 and 3.65, respectively^54,55^. Although the individual constituents of the ALP-AGS were not analyzed in the present study, EPS that was recovered from AGS contained mainly proteins (≈ 87%), polysaccharides (≈10%), and humic acids (2.3%)^56^. Proteins are the main source of carboxyl, hydroxyl, and amine groups, while polysaccharides and humic acids are the sources of hydroxyl groups^56^. Although the content of humic acids in the EPS from AGS was lower than that of proteins, humic acids are high in carboxylic acids and phenols, implying that they might be used as chelating agents. Humic acids and proteins can form complexes with cationic metals that are beneficial for metal adsorption^57^. The negative surface charge of EPS at a pH range of 3 to 10 is related to the deprotonation of carboxyl (pKa ≈ 3.0), phosphoryl (pKa ≈ 6.5), amine (pKa ≈ 8.4), and hydroxyl (pKa ≈ 10.2) groups^56^. In the EPS from the granular sludge, carboxyl and hydroxyl groups were most abundant and were mainly responsible for Pb^2+^, Cd^2+^, and Zn^2+^ adsorption^56^.

**Figure 6:**
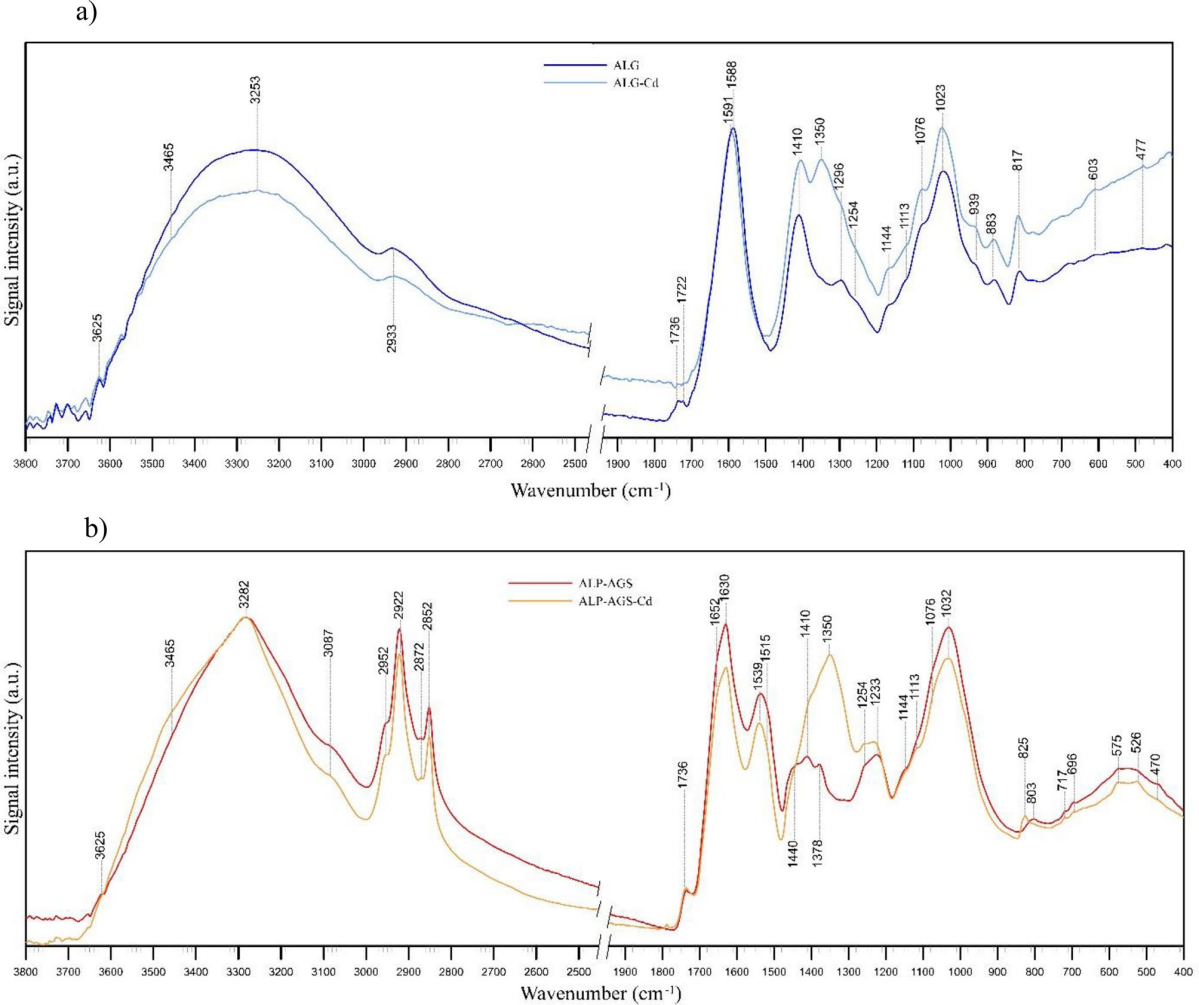
FTIR spectra of adsorbents before and after Cd^2+^ adsorption: (**a**) ALG, (**b**) ALP-AGS.

In the present study, all FTIR spectra (Fig. 6) contained absorption bands that indicated the presence of hydroxyl, ether, and carboxylic functional groups. From 3600 to 3000/cm, stretching vibrations of O–H bonds appeared, which are typical of polysaccharides^46^, and also N–H stretching vibrations of amino groups at ~ 3283/cm in the ALG-AGS spectra, which could confirm the presence of proteins^27,58,59^. At 2952–2852/cm in ALP-AGS spectra, stretching vibrations of aliphatic C–H were observed. These bands can be assigned to fatty acids^60^, and their intensity was greater in the ALP-AGS spectra than in the ALG spectra (Fig. 6b). An additional band at ~ 3087/cm in the ALP-AGS spectra indicates the presence of aromatic structures (also the band at ~ 1540/cm). The latter band may also have a contribution from the vibrations of amide II groups, i.e., N–H bending (also visible at 1515/cm) and C–N stretching in proteins^60^. The presence of C–N bonds is, in turn, confirmed by the band at 1378/cm^58^. The presence of hydrophobic components does not affect the adsorbent’s capacity for metal adsorption, but it can indirectly affect the location of polar hydrophilic groups responsible for metal adsorption^47^. The peaks at ~ 1730/cm are characteristic of C=O symmetric stretching in carboxylic acids. After Cd^2+^ adsorption, this band is no longer visible in the ALG spectrum (Fig. 6a), which may indicate that Cd^2+^ bonded with these acid groups. The bands at ~ 1590/cm (Fig. 6a) indicate the presence of ionic carboxylate salts.

From 1652 to 1630/cm in the ALP-AGS spectra, bands are visible that correspond to amide C=O and C–N stretching, N–H bending, C=C stretching in proteins, hydroxyl O–H stretching of polysaccharides^27,58,60^, and/or COO^–^ groups (Fig. 6b), which may indicate that these groups were more covalent in character. After Cd^2+^ adsorption, the intensity of these bands in the ALP-AGS spectra decreased (Fig. 6b), which may indicate the participation of carboxylate groups in the formation of complexes with Cd^2+^. The bands at 1100–1000/cm correspond to the glycosidic bonds in the polysaccharide (C–O–C stretching)^56,61,62^.

The presence of bands at ~ 1410/cm may result from the overlapping of C–H group vibrations and COO^–^ group stretching^61^. This band can be assigned to the stretching vibration of C=O and the deformation vibration of –OH in carboxylate, alcohol, or phenol structures^56,63^, and its enhancement after adsorption is explained by the rearrangement of molecular bonds and therefore the formation of new bands. After Cd^2+^ adsorption, both the ALG and ALP-AGS spectra have additional bands at ~ 1350/cm, and the bands at ~ 1410/cm have higher intensity, which may indicate both complexation of Cd^2+^ and the presence of nitrates (the source of the Cd^2+^ in the aqueous solution).

Thus, the high efficiency of Cd^2+^ removal at pH values over 4.0 was related to metal complexation with negatively charged functional groups (especially COOH and OH groups) on the ALG and ALP-AGS surfaces according to these reactions: 2ALG-COO^–^ + Cd^2+^ → (ALG-COO)_2_Cd^2+^ and 2ALP-AGS-COO^–^ + Cd^2+^ → (ALP-AGS-COO)_2_Cd^2+^^64^. The presence of monovalent species of Cd, which is due to the pH, can promote its complexation^65^. At pH < 6.0, it is present mainly as Cd^2+^. At pH > 6.0, the Cd^2+^ content gradually decreases and other Cd-containing species appear, e.g., CdOH^+^, Cd_2_OH^+^_3_, and Cd(OH)_2_(s)^51,66^. Due to the amphiphilic character of EPS from AGS and the presence of abundant negatively-charged functional groups, metals can also be adsorbed via electrostatic attraction, ion exchange, or surface precipitation^56,67^.

### Contact time and adsorption kinetics

**Figure 7:**
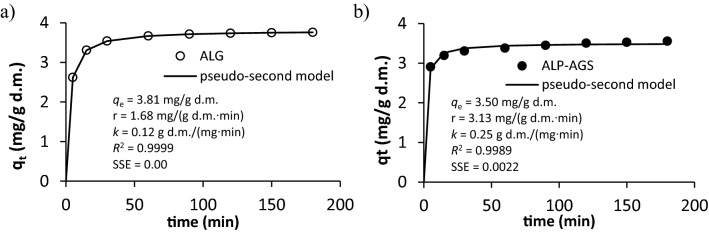
The effect on contact time on Cd^2+^ adsorption: (a) ALG, (b) ALP-AGS (10 mg Cd^2+^/L, pH 5.0; adsorbent dosage 2.6 g d.m./L). In the Figure, the values of parameters determined with pseudo-second kinetic order are included.

The effect of contact time on Cd^2+^ adsorption onto ALG and ALP-AGS is shown in Fig. 7. The amount of Cd^2+^ adsorbed at a specific adsorption time (*q*_t_) indicates that, as the contact time was increased from 5 to 60 min, Cd^2+^ adsorption increased, and then between 60 and 180 min, the adsorption curve was flat. Similarly, Liu et al.^56^ observed very quick metal (Pb^2+^, Cd^2+^, Zn^2+^) adsorption onto EPS recovered from AGS cultivated in a lab-scale sequencing batch reactor. The metal adsorption sharply increased within the first 10 min, and progressively slowed until saturation after about 60 min. The fast adsorption onto EPS could be related to metal interactions with functional groups of proteins. The R^2^ and SSE values indicate that Cd^2+^ adsorption onto both types of adsorbents at different contact times was well described by a pseudo-second-order kinetics model (Fig. 7)^54^.

The good fit of the model to the data indicates that the rate-limiting step in Cd^2+^ adsorption is chemisorption, which in the case of ALG and ALP-AGS, was due to complexation and electrostatic attraction between Cd^2+^ and negatively charged groups, as well as ion exchange between Cd^2+^ and other cations on the adsorbents’ surfaces, e.g., Ca^2+^. During the initial stage of adsorption, Cd^2+^ removal was rapid, as shown by the initial rate of adsorption (*r*), which was higher for ALP-AGS than for ALG. As time elapsed, the adsorption process slowed, due to the saturation of the available surface-active sites. For both adsorbents, equilibrium conditions for Cd^2+^ removal were reached within 60 min (Fig. 7).

### Initial Cd^2+^ concentration and adsorption isotherms

**Figure 8:**
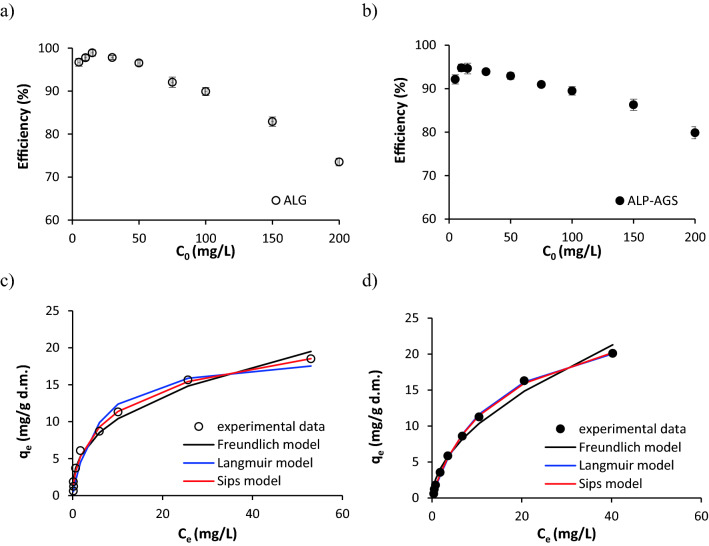
The efficiency of Cd^2+^ adsorption onto ALG and ALP-AGS depending on initial Cd^2+^ concentration (**a**, **b**) and isotherms of Cd^2+^ adsorption (**c**, **d**) (pH 5.0; 2 h; adsorbent dosage 7.9 g d.m./L) (n = 2). For ALP-AGS, the adsorption isotherm of the Langmuir model (blue line) coincides with the Sips model isotherm (red line).

The effects of the initial Cd^2+^ concentration (5–200 mg/L) on the metal removal efficiency and the adsorption capacity of the ALG and ALP-AGS are shown in Fig. 8a–d respectively.

The initial Cd^2+^ concentration affected the efficiency of adsorption with ALG and ALP-AGS. At initial Cd^2+^ concentrations in the range of 5–15 mg/L, the average Cd^2+^ removal efficiency slightly increased from 96.7 to 98.9% for ALG and from 92.1 to 94.6% for ALP-AGS. However, at initial concentrations above 15 mg/L, the efficiency gradually decreased to 73.5% (ALG) and 79.9% (ALP-AGS) (Fig. 8c, d). This means that the presence of available adsorption sites onto the adsorbents was high up to the relevant Cd^2+^ concentration.

The relationship between the amount of Cd^2+^ adsorbed by ALG and ALP-AGS and the concentration of Cd^2+^ remaining in the solution was evaluated with the Freundlich, Langmuir, and Sips models. Of these three adsorption models, the Sips model had the highest *R*^2^ values and the lowest SSE values (Table 2), indicating that it was the most suitable for describing Cd^2+^ adsorption onto tested adsorbents. The *q*_max_ is one of the principal criteria for the suitability of a particular adsorbent for metal removal. According to the Sips model, the *q*_max_ values were 29.3 mg/g d.m. for ALG and 29.5 mg/g d.m. for ALP-AGS. With comparable q_max_, a lower value for energy of adsorption (*b*) was for ALP-AGS. With the Langmuir model, the *q*_max_ value for ALP-AGS was similar to this calculated from the Sips model. Thus, ALP-AGS has the potential to serve as a substitute for commercial ALG. These *q*_max_ values are similar to those reported from other studies of Cd^2+^ adsorption with ALG adsorbents, e.g., ALG–calcium carbonate beads: *q*_max, Langmuir_ = 10.2 mg/g^53^, *q*_max, Langmuir_ = 37.8–68.9 mg/g^68^, *q*_max, Langmuir_ = 38.05 mg/g and *q*_max, Sips_ = 38.65 mg/g^47^. Liu et al.^56^ found that EPS extracted from AGS exhibited a high adsorption capacity for Cd^2+^ (1470 mg/g) that was much higher than for ALP-AGS and conventional biosorbents. The differences in adsorption capacities can be related to the methods used for the biopolymers’ recovery from the sludge matrix and their purification, which can provide more efficient exposure to the negatively charged sites. In the present study, ALP recovered from AGS was not purified.

**Table 2:** Parameters of Cd^2+^ adsorption onto ALG and ALP-AGS determined with selected adsorption models.

Adsorption model	Parameters	Unit	ALG	ALP-AGS
Freundlich	*K*_F_	L/g d.m.	4.3	2.9
	*n*	–	0.38	0.53
	*R*^2^	–	0.9893	0.9901
	*SSE*	-	2.858	5.134
Langmuir	*q*_max_	mg/g d.m.	19.4	26.9
	*K*_L_	L/mg	0.17	0.07
	*R*^2^	–	0.9837	0.9991
	*SSE*	–	0.992	0.160
Sips	*q*_max_	mg/g d.m.	29.3	29.5
	*b*	L/mg	0.16	0.07
	*n*	–	0.60	0.91
	*R*^2^	–	0.9964	0.9995
	*SSE*	–	0.475	0.0258

The Sips model being a combination of Langmuir and Freundlich isotherm models allows for predicting the heterogeneous adsorption systems. This model adjusts to low and high concentrations of metal ions where the interactions of the metal with the adsorbent are different^69^. Based on the values of heterogeneity index (n), adsorption of Cd^2+^ by ALP-AGS seems to occur onto more uniform and homogenous active sites compared to ALG^69^. A degree of homogeneity/heterogeneity of the adsorption sites can depend on the number of functional groups with the same capacity for adsorption. Cd^2+^ with low bonding strength is mainly adsorbed by carboxyl groups in the ALG molecule. Due to the conformational changes in the saccharide chains of G and M blocks, all carboxyl groups are not easily available, which affects the adsorption process^52^. Within the egg-box structure of ALG, the G-block carboxyl groups are less readily available to the metal ions, whereas the M-block carboxyl groups can more easily interact with Cd^2+^^70^. In contrast to ALG, active sites on ALP-AGS, come not only from polysaccharides but also from proteins or humic acids^71^. The investigations of metal adsorption onto individual EPS components (proteins, humic acids, and polysaccharides) recovered from different sludges have demonstrated that proteins exhibited the highest adsorption capacity for Cd^2+^ removal, while the polysaccharides were less efficient^71^. Thus, it might suggest that some irregularities in active sites onto polysaccharides in ALP-AGS can be compensated by active sites from other polymers co-existing in ALP.

### Chemical composition of alginate adsorbents after Cd^2+^ adsorption

SEM–EDX analysis of adsorbents after Cd^2+^ adsorption revealed that their composition had undergone some changes during the adsorption (Figs. 8, S2) process. Sorption of Cd^2+^ caused a decrease in the share of most elements analyzed with SEM–EDX, especially C (which may be related to changes in the carbon structure of alginate), as well as Ca (which may be related to the ion exchange mechanism of Cd^2+^ adsorption)^72^. Alkali metals play the role of ion exchange in the process of metal adsorption. Among them, Ca ions play an important role in the ion exchange of metal cations under medium acidic conditions^73,74^. In the present study, the experiment on Cd adsorption was performed at pH 5.0, and Ca release was observed from both ALG and ALP-AGS. Similarly, Ablough et al.^75^ found that after the adsorption of Pb on hybrid beads of chitosan and sodium alginate, the Pb peak appeared in the EDX spectra, while the Ca peak disappeared, indicating that Ca was completely removed from the adsorbent and ion exchange was the main mechanism of Pb adsorption. Bée et al.^76^ related the binding of Pb to Ca-alginate beads to the release of Ca and found that the amount of Ca remaining in the biopolymer bead was very small when the sorbent was saturated with Pb. Some divalent heavy metal ions with high reactivity can exchange Ca ions while maintaining the structure of the crosslinked adsorbent^55^ without unraveling the polymer.

**Figure 9:**
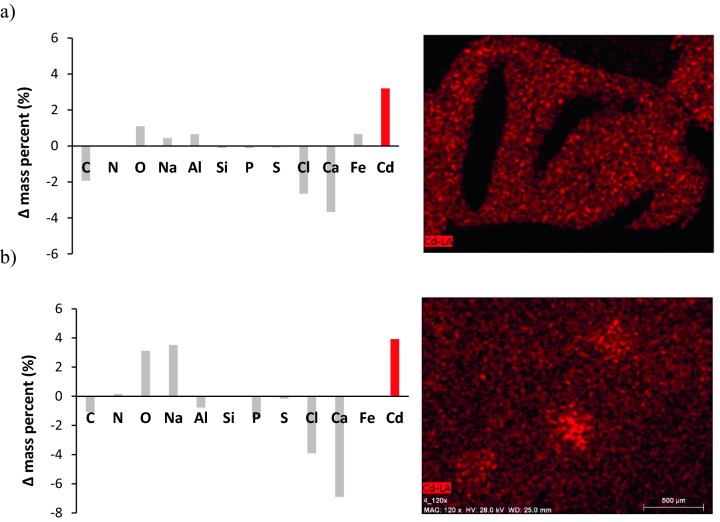
Changes in the chemical composition of ALG (**a**) and ALP-AGS (**b**). Δ mass percent indicates the difference in the content of an individual element before and after Cd^2+^ adsorption (a positive value = an increase of a given element, a negative value = a decrease of a given element). The images show the elemental mapping of Cd after adsorption on ALG and ALP-AGS surface (pH 5.0, adsorbent dosage 7.9 g d.m./L, 200 mg Cd^2+^/L, 2 h).

The surface EDX mapping of the ALG and ALP-AGS proved the realization of the Cd^2+^ adsorption process, with comparable mass % of Cd^2+^ onto ALP-AGS (3.92 ± 1.25 mass %) and ALG surfaces (3.20 ± 0.43 mass %). These results correspond well with the maximum adsorption capacities estimated from Langmuir and Sips models (Table 2). The images obtained through mapping elements in SEM–EDX for Cd^2+^ exhibit uniform metal distribution on the surface of both adsorbents (Fig. 9).

## Conclusions

This study showed that AGS from a full-scale WWTP is a rich source of ALP, which can be used for the effective adsorption of Cd^2+^. ALP content in the AGS from the full-scale facility increased in the transition period between winter and spring, reaching over 150 mg/g MLSS. In the batch reactor cycle, ALP content was highest 1 h after the start of aeration, about 2 times higher than at the end of the cycle. ALP-AGS has a low total pore volume and surface area, and its sorption properties were determined by the presence of carboxylic and hydroxyl groups. The Cd^2+^ removal mechanism was governed by chemisorption and a monolayer of Cd^2+^ on ALP-AGS surface was created. The most efficient Cd^2+^ removal was observed at an adsorbent dosage of 7.9 g d.m./L. ALP-AGS, pH ranging from 4 to 8, and 60 min. Under these conditions, the mass percentage of Cd^2+^ adsorbed onto ALP-AGS was 3.92%, while that adsorbed onto commercial ALG was 3.20%. ALP-AGS can serve as an attractive substitute for commercial ALG in the elimination of toxic Cd^2+^ from the environment, and sustainable ALP recovery from waste AGS can be implemented as part of comprehensive strategies for utilizing wastewater in a circular economy.

## Supplementary Information

Supplementary Figures.

## Data Availability

All data generated or analyzed during this study are included in this published article and its supplementary information files.
