# Supplementary material for: Alginate-like polymers from full-scale aerobic granular sludge: content, recovery, characterization, and application for cadmium adsorption
Source: Sci Rep. 2022 Dec 23;12:22260. doi: 10.1038/s41598-022-26743-5 (PMC9789099; doi:10.1038/s41598-022-26743-5)
Supplement: Supplementary file 1 — Supplementary Figures. [file 41598_2022_26743_MOESM1_ESM.docx]

**Supplementary Material**

**Alginate-like polymers from full-scale aerobic granular sludge: content, recovery, characterization, and application for cadmium adsorption**

Agnieszka Cydzik-Kwiatkowska, Mariusz Z. Gusiatin*, Magdalena Zielińska, Irena Wojnowska-Baryła, Dorota Kulikowska, Katarzyna Bernat

Department of Environmental Biotechnology, Faculty of Geoengineering, University of Warmia and Mazury in Olsztyn, Słoneczna St. 45G, 10-709 Olsztyn,

*Corresponding author: mariusz.gusiatin@uwm.edu.pl

**Fig. S1**. Wastewater temperature in full-scale GSBRs during the experiment (n = 2)

Mapping of ALG


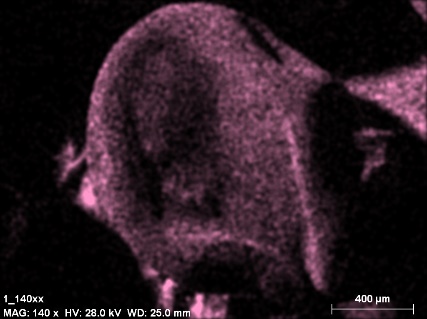

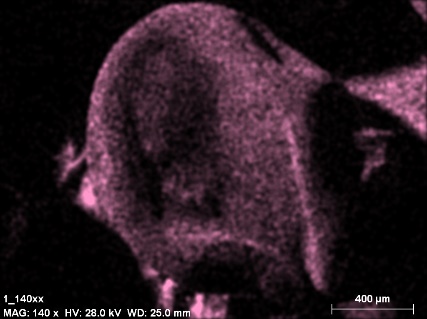

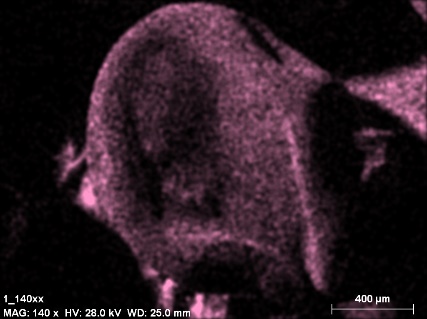

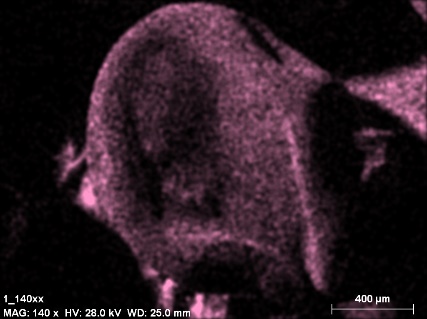

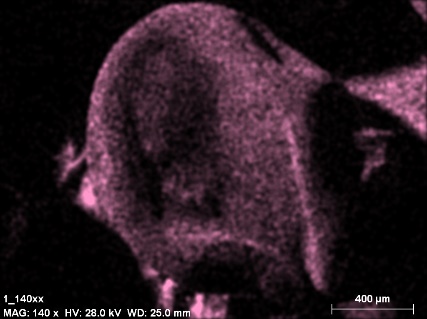

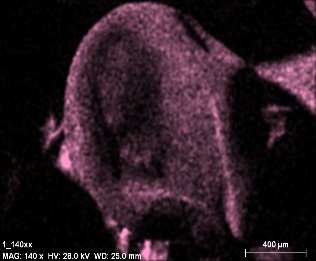

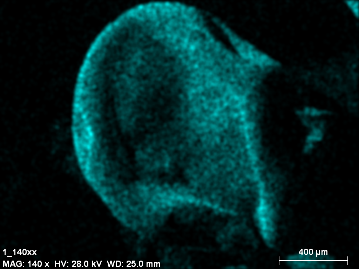

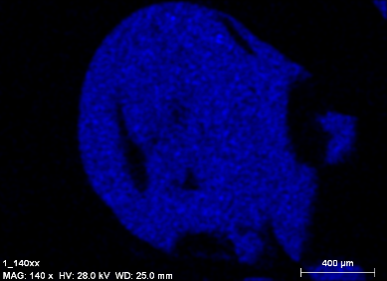

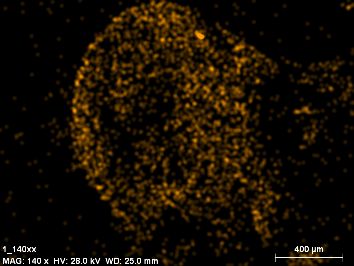

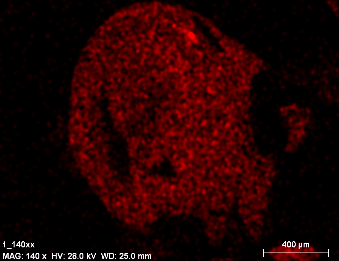


Cl

Na

Ca

O

C

Mapping of ALP-AGS

P


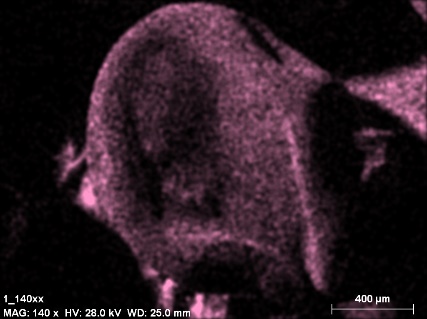

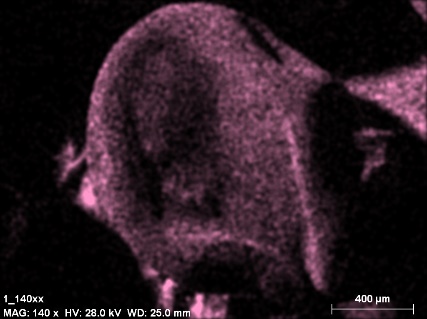

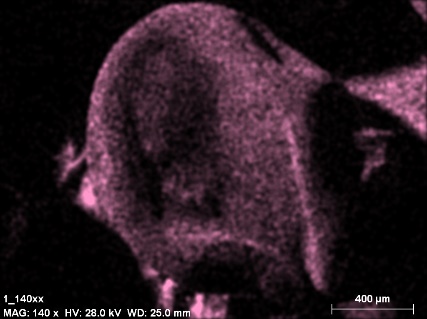

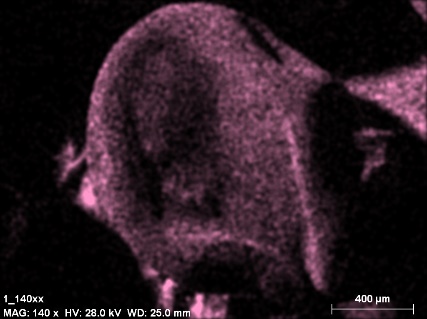

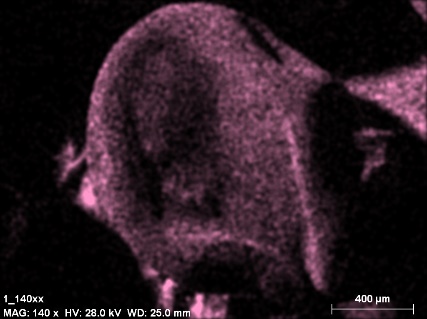

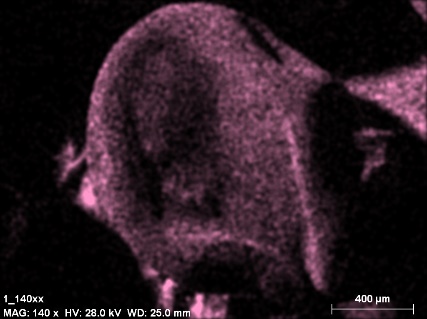

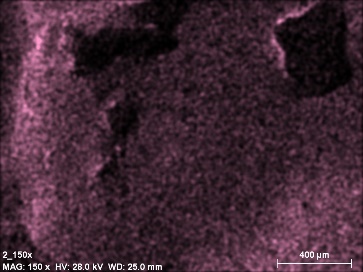

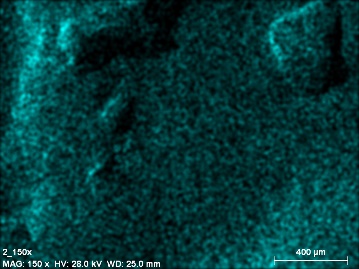

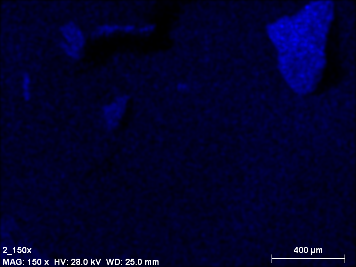

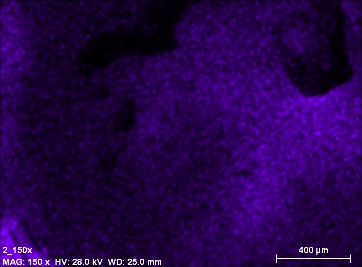

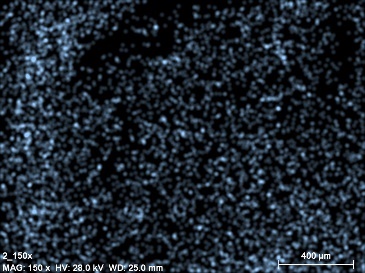

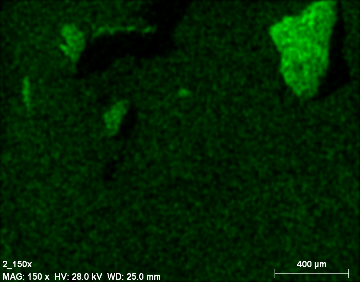


N

Cl

Ca

O

C

**Fig. S2**. Chemical composition and exemplary elemental mapping of ALG and ALP-AGS before adsorption experiments
